# Editorial: Rising stars in insect physiology

**DOI:** 10.3389/finsc.2024.1483760

**Published:** 2024-09-26

**Authors:** Peter M. Piermarini, Nicholas M. Teets

**Affiliations:** ^1^ Department of Entomology, College of Food, Agricultural, and Environmental Sciences, The Ohio State University, Wooster, OH, United States; ^2^ Department of Entomology, University of Kentucky, Lexington, KY, United States

**Keywords:** insect, physiology, toxicology, molecular, biochemistry, early career

We are excited to present the inaugural Frontiers in Insect Science ‘Rising Stars in Insect Physiology’ Research Topic. When envisioning this Research Topic in 2022, our goal was to highlight the recent research contributions of early career investigators (graduate students, postdoctoral researchers, assistant professors) in Insect Physiology, with an emphasis on research topics that are integrative and/or multidisciplinary in nature. We aligned this Research Topic with a new travel award opportunity in 2022 that was co-sponsored by Frontiers and the Physiology, Biochemistry, and Toxicology (PBT) section of the Entomological Society of America (ESA), which sought to enhance the diversity of PBT section membership by supporting the travel of graduate students from under-represented groups to the annual conference of ESA (PBT Graduate Student Travel Award). Based on their impressive credentials, several applicants to this travel award were invited as contributors to the current Research Topic, and we were pleased that one of the inaugural awardees of the PBT Graduate Student Travel Award (Nia I. Keeys-Scott) was the primary author on the first manuscript to be accepted for publication in this Research Topic (Keyes-Scott et al.).

The four original contributions in this Research Topic focus on molecular and/or biochemical insights into the physiology of mosquitoes. Keyes-Scott et al. demonstrated the role of two previously orphaned G protein-coupled receptors (GPCRs) in reproduction of *Aedes aegypti*. Bianco et al. found that diapause in *Culex pipiens* can be disrupted by feeding them royal jelly produced by honey bees (*Apis mellifera*), which is enriched with Major Royal Jelly Protein 1, or by knocking down the mRNA encoding the orthologous protein in *C. pipiens*. Picinic et al. characterized the localization of several aquaporin (AQP) proteins in the alimentary canal, fat body, and ovaries of *A. aegypti* and demonstrated that localization was impacted by blood feeding, providing insights into putative roles in water and/or metabolite transport in these tissues. Finally, Sajadi and Paluzzi characterized the molecular and immunochemical expression of an understudied insect neuropeptide (ion transport peptide, ITP) in *A. aegypti* and used RNAi to uncover putative roles in excretory physiology, reproduction, and blood feeding.

The six reviews/mini-reviews in this Research Topic cover a variety of topics with connections to Insect Physiology and Toxicology. Abendroth et al. review recent evidence suggesting a non-canonical role of odorant binding proteins (OBPs) in adaptation of insects to xenobiotics. Mack and Attardo discuss the relationship between thermotolerance and insecticide resistance in mosquitoes. They uncover a novel intersection between these physiological pathways that may be mediated by heat shock proteins, which has potential implications for vector control in the current era of climate change. Luker provides a critical review on the laboratory assays used in the past two decades to discover and assess the efficacy of mosquito repellents. Weger and Rittschof review the diverse physiological roles of insulin and insulin-like growth factor signaling in adult insects, along with molecular and neural mechanisms connecting insulin signaling to nutrition and behavior. Dates and Kolosov review novel and emerging roles of voltage-gated ion channels in non-excitable tissues, such as epithelia, where these channels have been understudied and may play key roles in epithelial transport and cell signaling. Finally, Vinauger and Chandrasegaran review studies on *A. aegypti* that use laboratory, semi-field, and field experiments to elucidate potential interactions between mosquito physiology and behavior and highlight the complications of studying mosquito life history traits associated with variations in larval competition nutrition and competition.

Together, these articles reflect that the future of insect physiology is in good hands, with a talented and diverse group of early career scientists leading the way. Insect physiology research is becoming increasingly integrative, and the above papers highlight that trend. The primary studies in this Research Topic incorporate approaches such as organismal biology, organ physiology, molecular biology, and behavior to demonstrate the complex interplay of factors that shape insect function. The review articles also synthesize diverse topics to highlight the importance of looking across traditional disciplinary boundaries (e.g., endocrinology, nutrition, behavior, etc.) to reveal insights into insect biology. We also want to thank an excellent group of reviewers for providing timely, thorough comments in support of these early career scientists. Through the efforts of the authors and review community, these 10 papers showcase some of the rising stars in our discipline that will lead the field into the next quarter of the century.

